# Assessment of Exclusive Breastfeeding Practice and Associated Factors among Mothers in West Shoa Zone, Oromia, Ethiopia

**DOI:** 10.1155/2020/3965873

**Published:** 2020-08-12

**Authors:** Kassa Mamo, Tizita Dengia, Abuzumeran Abubeker, Eden Girmaye

**Affiliations:** ^1^Department of Midwifery, College of Medicine and Health Sciences, Ambo University, Ambo, Ethiopia; ^2^Department of Public Health, College of Medicine and Health Sciences, Ambo University, Ambo, Ethiopia

## Abstract

**Background:**

The World Health Organization (WHO) recommends mothers worldwide to exclusively breastfeed infants for the child's first six months to achieve optimal growth, development, and health. Even though appropriate feeding practice is the most cost-effective intervention to reduce child morbidity and mortality, exclusive breastfeeding practices in developing countries are still low.

**Objective:**

The objective of the study was to assess exclusive breastfeeding practice and associated factors among mothers in West Shoa zone.

**Methods:**

Community-based cross-sectional study design was conducted from May to December 2018 in the West Shoa zone, Ethiopia, among 710 mothers with 6–9-month-old infants. The multistage stage sampling technique was employed. A pretested structured interviewer-administered questionnaire was used to collect the data. Epi Info version 7.1.2.0 was used to enter the data, and we transferred to SPSS version 25 for analysis. The association between factors and the exclusive breastfeeding were analyzed with bivariate and multivariate logistic regression.

**Result:**

A total of 710 women were included with a response rate of 97.9%. The prevalence of unintended pregnancy was 38.7%, and only 65.35% of the respondents reported that they have exclusively breastfed for the first six months of their infant's life. Marital status (AOR 2.467 (1.333–4.564)), ANC visit (AOR 2.562 (1.250–5.252)), pregnancy intentionality (AOR 4.727 (3.217–6.945)), postnatal care clinic attendance (AOR 3.373 (2.293–4.963)), and counseling on exclusive breastfeeding AOR 2.544 (1.239–5.225) were the factors associated with exclusive breastfeeding. Exclusive breastfeeding practice is still low and actions need to be taken like educating the community about the importance of exclusive breastfeeding using every accessible media. Maternal health service centers should provide counseling and education for women about breastfeeding.

## 1. Introduction

The World Health Organization recommends mothers to exclusively breastfeed infants for the child's first six months of life to achieve optimal growth, development, and health [1]. Babies who are breastfed exclusively for 6 months experience fewer illnesses because breast milk contains nutrients and substances that protect the baby from several infections and major childhood conditions, diarrhea, gastrointestinal tract infection, allergic diseases, diabetes, obesity, childhood leukemia and lymphoma, and inflammatory and bowel disease and it leads to improved cognitive development [2, 3].

According to Black et al. [4], suboptimal breastfeeding especially nonexclusive breastfeeding in the first six months of life results in 1.4 million deaths and 10% of disease burden in children younger than 5 years [5]. However, only 35% of infants worldwide are exclusively breastfed during the first four to six months of life and complementary feeding begins either too early or too late with foods that are often nutritionally inadequate and unsafe [6, 7].

Notwithstanding the fact that appropriate feeding practice is the most cost-effective intervention to reduce child morbidity and mortality [6], exclusive breastfeeding practices in developing countries are still low. Of approximately 56 million infants less than six months of age in developing countries, approximately 22 million are exclusively breastfed, while over 34 million children (60.7%) are not. Eighty percent of these children who do not benefit from exclusive breastfeeding in developing countries live only in 29 countries. From these 29 countries, the 10 large countries including Ethiopia have two-thirds (over 21 million) of the approximate numbers of nonexclusively breastfed children in developing countries [8].

In Ethiopia, breastfeeding is a common practice, but a large proportion of mothers do not practice optimal breastfeeding. According to EDHS 2016 estimated, 58% of under 6-month aged infants are exclusively breastfed. It is estimated that 74.1% of 0-1-month-old infants, 64% of 2-3-month-old infants, and 36% of 4-5 infants were exclusively breastfed [9].

## 2. Methods

### 2.1. Study Design and Setting

A community-based cross-sectional study design was employed. The study was conducted in west Shoa zone, Oromia, Ethiopia, from April to December 2018. West Shoa zone is one of the zones of the Oromia regional state. Ambo town is the capital of West Shoa. The zone consists of 22 districts and 583 kebeles. According to the 2007 Census conducted by the Central Statistics Agency of Ethiopia (CSA), this zone has a total population of 2,058,676, of whom 1,028,501 are men and 1,030,175 women, with an area of 14,788.78 square kilometers.

### 2.2. Sample Size Determination

All mothers whose infants are 6–9 months old and whose permanent residents in the area are for at least 6 months prior to conception were included in the study and mothers who are mentally disabled and unable to respond were excluded.

The sample size was calculated by using single population proportion formula using the prevalence of exclusive breastfeeding 34.1% from the study conducted in Bishoftu [10], 95% confidence interval, and 5% of margin error:

(1) $$\begin{array}{c} n=\frac{{\left(Z\left(\alpha /2\right)\right)}^{2}P\left(1-P\right)}{d^{2}},\\ n_{i}=\frac{{\left(1.96\right)}^{2}\;\left(0.341\right)\left(1-0.341\right)}{{\left(0.05\right)}^{2}}=345. \end{array}$$

Considering design effect and 10% nonresponse rate, the final sample size is 725.

### 2.3. Data Collection Instrument and Sampling Procedures

A pretested structured interviewer-administered questionnaire was used to collect the data. Multistage sampling was used assuming homogeneity of the population in the study area. Twenty percent of the 22 districts, that is, 5 of them, were randomly selected using lottery method. Then, 20% of the kebeles in the districts were randomly selected using lottery method based on their number in order to ensure the representativeness of data. A structured questionnaire was developed after reviewing different literature in English language and translated into the local language. The questionnaire includes three parts which elicit information on the sociodemographic characteristics of the study participants, obstetric characteristics, and exclusive breastfeeding practice. A pretested structured interviewer-administered questionnaire was used to collect the data. The data were collected by face-to-face interviews by trained data collectors who were BSc midwives and fluent in the local dialect.

### 2.4. Data Quality and Analysis

The questionnaire was developed in English and translated into the local language Afan Oromo. Pretest was made on 5% (37 mothers) of the total sample size. Training was provided to the data collectors on how to collect the data. All questionnaires were checked for completeness, coded, and entered using Epi Info version 7.1.2.0 and transferred to SPSS version 25 software package for analysis. Descriptive statistics such as mean, percentage, and standard deviations were determined. Data were analyzed using SPSS version 25 statistical software. The possible association was observed with binary logistic regression. All variables which have less than a *p* value of 0.05 in the binary logistic regression were entered to multivariate logistic regression model. *p* values of ≤0.05 were used to declare significant association. Crude and adjusted odds ratios with their 95% confidence intervals were determined.

## 3. Results

### 3.1. Sociodemographic Characteristics of the Study Participants

A total of 710 women were included in the study, making a response rate of 97.9%. The age of the respondents ranged from 16 to 43 years with the mean and median age being 28.9 (±6.04) and 29 years, respectively. Majority of the respondents (73.7%) were Oromo by ethnicity. Most of the respondents were married (90.1%). Urban dwellers make up 56.8% of the total respondents (Table 1).

### 3.2. Obstetric Characteristics of Respondents

More than 58% of women have at least one ANC visit in their most recent pregnancy. 36.2% the respondents are primiparous. Majority of the women have given birth in health institutions while 644 (90.7%) women gave birth vaginally. Among the respondents, 275 (38.7%) women reported that their most recent pregnancy was unintended. Among them, 128 (46.5%) women said that their pregnancy was mistimed and the rest 147 (53.5%) have had completely unwanted pregnancy (Table 2).

### 3.3. Exclusive Breastfeeding Practice

**Figure 1:**
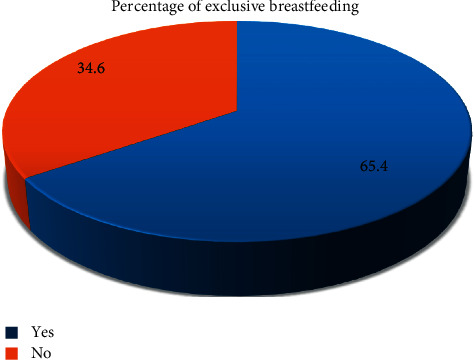
Percentage of exclusive breastfeeding among mothers in west Shoa zone.

In this study the percentage of women who have exclusively breastfed accounts for 65.4% (464); the remaining 246 women reported that they did not breastfed exclusively (Figure 1). Among mothers who did not exclusively breastfed their infants for a minimum of 6-month duration, 48% (118) said that they never exclusively breastfed their child (Table 3).

### 3.4. Bivariate and Multivariate Analyses of Factors Associated with Exclusive Breastfeeding

On bivariate analysis besides intention of pregnancy, multiple factors were sought for possible association with exclusive breastfeeding including age, marital status, place of residence, educational level, monthly income, ANC visit, mode of delivery, place of delivery, and sex of the child. Multivariate analysis identified that factors significantly associated with exclusive breastfeeding were intention of pregnancy, marital status, ANC visit, PNC attendance, and counseling on EBF. Women with intended pregnancy were more than four times likely to breastfeed exclusively compared to their counterparts (AOR 4.727, 95% CI (3.217–6.945). Exclusive breastfeeding practice was higher among married women than single AOR 2.467, 95% CI (1.333–4.564)). Mothers who have ANC follow-up were 2.562 more likely to breastfeed their child compared to women who did not have follow-up (AOR 2.562, 95% CI (1.250–5.252)). Mothers who have PNC attendance were more than 3 times likely to exclusively breastfeed their babies compared to mothers who did not have PNC attendance (AOR 3.373, 95% CI (2.293–4.963)) (Table 4).

## 4. Discussion

The purpose of the study was to assess exclusive breastfeeding practice and associated factors. Among the study participants, 464 (65%) have reported that they have exclusively breastfed their babies for the appropriate time. The finding indicated that the prevalence of EBF is comparable to a study conducted in Debre Markos town which is 60.8% [11] but greater than studies in other parts of the country such as Bahirdar (50.3%) [12], Mecha (47.13%) [13], Injibara (44%) [14], and Axum (40.9%) [15]. Compared to the studies conducted in different parts of the world, the result showed that it is higher, where only 26% of the mothers in Cameroon [16], 20% in Nigeria [17], 48% in Ghana [18], 44.3% in Malaysia [19], and 36% in Bangladesh [16] reported that they have exclusively breastfed their babies. Considering the time these studies conducted, it would make sense for the variations to be huge as the institutional delivery and health-seeking behaviors have been increasing over time. Yet, it is lower than a study conducted in Saudi Arabia (76.1%) [20].

38.7% of the pregnancies in the current study were unintended. Among them, 128 (46.55%) women stated that their pregnancy was mistimed and 147 (53.45%) women reported it was unwanted. It is comparable to studies in Ethiopia like Hosanna (34%) [21], Ganji (36.5%) [22], Harar (33.3%) [23], and Damot Gale (42.4%) [24]; however, it is higher than studies in Kersa (27.9%) [25], Dessie (21.5%) [26], and Gelemso (27.1%) [27] but less than the finding from Adigrat (69.5%) [28]. It is less than a study conducted in Brazil which was 55.4% [29].

Pregnancy intentionality was independently associated with exclusive breastfeeding. The odds of EBF among women who have intended pregnancy were four times higher than the odds in women who have unintended pregnancy (AOR 4.727, 95% CI (3.217–6.945)). In line with the current study, a cross-sectional study from Hosanna has reported that planned pregnancies were more likely to be breastfed than their counterparts [21]. Similar findings were reported from other studies too, where unintended pregnancy does not only affect breastfeeding but also the prenatal care uptake during their pregnancy, and when the pregnancy is unwanted or mistimed, they either will initiate untimely or will not continue breastfeeding [30, 31]. Unintended pregnancy has also been associated with adverse pregnancy outcomes [32, 33]; correspondingly, reports indicated that timely wanted pregnancies are more likely to lead to breastfeeding initiation and longer duration [34]. This implies the importance of the provision of effective contraception to prevent unintended pregnancy which in turn affects child health and development.

The odds of EBF among women who did get counseling on exclusive breastfeeding were two times higher than the odds in women who did not get counseling on exclusive breastfeeding (AOR 2.544, 95% CI (1.239–5.225)). In line with other studies, women who have ANC follow-up (AOR 2.562, 95% CI (1.250–5.252)) and PNC (AOR 3.373, 95% CI (2.293–4.963)) follow-up were more likely to exclusively breastfeed than their counterparts [13] [27]. Different studies reported that counseling on exclusive breastfeeding and infant feeding during their ANC follow-up and PNC follow-up has a strong association with exclusive breastfeeding [11–13, 35, 36], which indicates the importance of effective counseling during prenatal and postnatal follow-up.

Marital status was found to be associated with EBF; married mothers were more than two times likely to exclusively breastfeed than single mothers (AOR 2.467, 95% CI (1.333–4.564)). A similar finding has reported that nonexclusive breastfeeding was more likely to be practiced by mothers who were not married than married counterparts (AOR (95% CI) = 2.6 (1.1, 6.0)) [37] that might be related to the fact that responsibility-sharing among family members could help the practice of exclusive breastfeeding.

## 5. Conclusion

64.5% of women have practiced exclusive breastfeeding. ANC follow-up, PNC attendance, counseling, and pregnancy intentionality were factors associated with exclusive breastfeeding. Mothers should be counseled on the importance of exclusive breastfeeding during their ANC follow-up and postnatal visits. The study might suffer from limitations like information and recall biases to different variables which may result in under- or overestimation of exclusive breastfeeding practice. The nature of the study design could prevent an establishment of a cause-effect relationship between the factors and the dependent variable (EBF).

## Figures and Tables

**Table 1:** Sociodemographic characteristics of the study participants among women in west Shoa zone.

		Frequency	Percent
Age of the mother	<18	44	6.2
	18–35	532	74.9
	>35	134	18.9
	Total	710	100.0

Ethnicity	Oromo	498	70.14
	Amhara	178	25.1
	Gurage	25	3.52
	Others	9	1.3
	Total	710	100.0

Marital status	Married	640	90.1
	Single	70	9.9
	Total	710	100.0

Religion	Orthodox	242	34.1
	Protestant	433	61.0
	Muslim	35	4.9
	Total	710	100.0

Residence	Urban	403	56.8
	Rural	307	43.2
	Total	710	100.0

Educational status	Illiterate	138	19.4
	Can read and write with no formal education	82	11.5
	Completed elementary/grades 1–8	153	21.5
	Completed secondary school/grades 9–12	184	25.9
	Above grade 12	153	21.5
	Total	710	100.0

Monthly income	Very low (<600)	181	25.5
	Low (601–1500)	128	18.0
	Average (1501–3500)	167	23.7
	Above average (3501–5000)	168	9.3
	High (>5001)	66	30.1
	Total	710	100.0

**Table 2:** Obstetric characteristics of the study participants among women in west Shoa zone.

Variables		Frequency	Percent
ANC visit	Yes	432	60.8
	No	278	39.2

Parity	Primiparous	257	36.2
	Multiparous	453	63.8

Intention of pregnancy	Wanted	435	61.3
	Mistimed	128	38.7
	Unwanted	147	

PNC attendance	Yes	441	62.1
	No	269	37.9

Place of delivery	Home	199	28.0
	Health institution	511	72.0

Mode of delivery	Vaginal delivery	644	90.7
	Cesarean delivery	66	9.3

**Table 3:** Duration of exclusive breastfeeding among women in west Shoa zone.

Duration of exclusive breastfeeding	Frequency	Percent
Never exclusively breasted	118	48
Less than 1 month	11	4.5
Between 1 and 2 months	100	40.6
Between 3 and 5 months	17	6.9
Total	246	100

**Table 4:** Bivariate and multivariate regression analysis of factors associated with exclusive breastfeeding among women in west Shoa zone.

Variables		Exclusive breastfeeding		COR (95% CI)	AOR (95% CI)	*p* value
		Yes (%)	No (%)			
Marital status	Married	440 (62.0%)	200 (28.2%)	4.217 (2.504–7.100)	2.467 (1.333–4.564)	0.004
	Single	24 (3.4%)	46 (6.5%)	1.00	1.00	

Residence	Urban	321 (45.2%)	86 (12.1%)	4.176 (3.009–5.797)	^*∗∗*^	
	Rural	143 (20.1%)	160 (22.5%)	1.00		

ANC visit	Yes	348 (49.0%)	84 (11.8%)	5.786 (4.130–8.105)	2.562 (1.250–5.252)	0.010
	No	116 (16.3%)	162 (22.8%)	1.00		

Intention of pregnancy	Intended	349 (49.2%)	86 (12.1%)	5.646 (4.034–7.903)	4.727 (3.217–6.945)	0.000
	Unintended	115 (16.2%)	160 (22.5%)	1.00		

PNC attendance	Yes	337 (47.5)	104 (14.6%)	3.623 (2.618–5.015)	3.373 (2.293–4.963)	0.000
	No	127 (17.9%)	142 (20.0%)	1.00		

Mode of delivery	Home	432 (60.8)	212 (29.9%)	2.165 (1.300–3.605)	^*∗∗*^	
	Institutional	32 (4.5%)	34 (4.8%)	1.00		

Counseling on EBF	Yes	355 (50.0%)	90 (12.7%)	5.645 (4.031–7.905)	2.544 (1.239–5.225)	0.011
	No	109 (15.4%)	156 (22.0%)	1.00		

Sex of the child	Male	311 (43.8%)	138 (19.4%)	1.519 (1.162–1.985)	^*∗∗*^	
	Female	151 (21.3%)	104 (14.6%)	1.00		

No significant association on multivariate regression analysis.

## Data Availability

The data used in this study are available on request from the corresponding author.

## Abbreviations

AOR: Adjusted odds ratio
DHS: Demographic and health survey
CI: Confidence interval
EDHS: Ethiopian demographic and health survey
EBF: Exclusive breastfeeding
NGOs: Nongovernmental organizations
SSA: Sub-Saharan Africa
WHO: World Health Organization.

## Supplementary Materials

Supplementary Materials The questionnaire used in this study.
