# Supplementary material for: Assessment of Exclusive Breastfeeding Practice and Associated Factors among Mothers in West Shoa Zone, Oromia, Ethiopia
Source: Obstet Gynecol Int. 2020 Aug 12;2020:3965873. doi: 10.1155/2020/3965873 (PMC7441431; doi:10.1155/2020/3965873)
Supplement: Supplementary Materials — The questionnaire used in this study. [file 3965873.f1.docx]

# Questionnaire

# Consent

**Code No. _______________**

Hello! My name is ………………………………….. I am here to collect information on **“Assessment of exclusive breastfeeding practice and associated factors among mothers in West Shoa Zone, Oromia, Ethiopia”**. The main part of the study involves collecting information from you on your pregnancy and your breastfeeding practice. You were selected because you have infant. In accepting to participate in the study, you are allocating to us about 40 minutes of your time to answer the questions. There is no risk associated with participating in this study. It involves no treatment or procedure that could cause harm, injury or discomfort to you or your child. It involves collecting information by answering questions. The answers to the questions will be recorded. Your response will benefit the Ambo town health bureau to plan more effective interventions to promote mothers’ and infants’ health in your community. Participation in this study is entirely voluntary. You are perfectly free to withdraw from the study at any time – even in the middle of the interview.

We assure all information gathered during the course of the study will be kept completely confidential. Only the principal investigators and the research assistants collecting the data will have access to the data. All the data will be coded for identification.

Would you be willing to participate?

Yes …….1 No ……….2

Having been well explained and informed of the intentions and benefits of the study, I voluntarily consent to participate in the study.

 Interviewer name ________________ Sign. _______________ Date ___________

**Instructions:** Show your answer by circling the listed choices. For open-ended questions, give short and precise answer.

**Part I. Socioeconomic and demographic characteristics of respondents**

| S. No. | Questions | Response | Remark |
| --- | --- | --- | --- |
| 101 | How old are you? (in years) | _________________ |  |
| 102 | To which ethnic group do you belong? | 1.Oromo 2. Amhara 3. Gurage 4. Wolayta  5. Others (specify)____ |  |
| 103 | What is your marital status? | 1.Married 2. Single |  |
| 104 | What is your religion? | 1. Orthodox 2. Protestant 3. Muslim 4.Others(specify) ____ |  |
| 105 | What is your educational level? | 1. Illiterate 2. Read and write but with no formal education 3. Elementary/ Grade 1-8 4.Secondary school/ Grade 9-12  5. Above grade 12 |  |
| 106 | Residence | 1. Urban 2. Rural |  |
| 107 | What is your monthly family income? (birr/month) for urban residents | ___________________ |  |

**Part II. related questions**

| S.N. | **Questions** | **Responses** | **Remark** |
| --- | --- | --- | --- |
| 201 | How many child/children do you have? | 1.1 2. 2 3. 3 4. Specify no. ____ |  |
| 202 | At the time you became pregnant, | 1. *I want to be pregnant by then*  2. *I want to wait some time*  3. *I don’t want to get pregnant all.* |  |
| 203 | Did you attend ANC? | 1. Yes 2. No |  |
| 204 | If your answer for question 208 is “yes” how many visits do you have? | 1.One visit 2. Two visits  3. Three visits 4. Four and above visits |  |
| 205 | Did you attend PNC? | 1. Yes 2. No |  |
| 206 | If your answer for question 208 is “yes” how many visits do you have? | 1. One visit 2. Two visits  3. Three and above visits |  |
| 207 | Where did you give birth/deliver? | 1. At home 2. Health institution |  |
| 208 | What was the mode of delivery? | 1. Vaginal delivery 2. Cesarean delivery |  |

**Part III. Exclusive breastfeeding related questions**

| **S.N.** | **Items** | **Responses** | **Remark** |
| --- | --- | --- | --- |
| 301 | What is the sex of your child? | 1. Male 2. Female |  |
| 302 | Did you exclusively breastfeed your child for six months? | 1. Yes 2. No |  |
| 303 | If the answer for question 304 is no, how long did you exclusively breastfeed your child? | 1. Never exclusively breastfed 2. Less than 1 month 3. Between 1 – 2 months 4. Others (specify)______________ |  |
| 304 | Have you received counseling on EB during your pregnancy or delivery? | 1. Yes 2. No |  |
